# Increased reporting but decreased mortality associated with adverse events in patients undergoing lung cancer surgery: Competing forces in an era of heightened focus on care quality?

**DOI:** 10.1371/journal.pone.0231258

**Published:** 2020-04-09

**Authors:** Mitchell S. von Itzstein, Arjun Gupta, Kemp H. Kernstine, Kristin C. Mara, Sahil Khanna, David E. Gerber

**Affiliations:** 1 Department of Internal Medicine, UT Southwestern Medical Center, Dallas, TX, United States of America; 2 Department of Cardiothoracic Surgery, UT Southwestern Medical Center, Dallas, TX, United States of America; 3 Department of Health Sciences Research, Mayo Clinic, Rochester, MN, United States of America; 4 Division of Gastroenterology, Mayo Clinic, Rochester, MN, United States of America; 5 Department of Population & Data Sciences, UT Southwestern Medical Center, Dallas, TX, United States of America; 6 Harold C. Simmons Comprehensive Cancer Center, UT Southwestern Medical Center Dallas, TX, United States of America; Auburn University, UNITED STATES

## Abstract

**Introduction:**

Advances in surgical techniques have improved clinical outcomes and decreased complications. At the same time, heightened attention to care quality has resulted in increased identification of hospital-acquired adverse events. We evaluated these divergent effects on the reported safety of lung cancer resection.

**Methods and materials:**

We analyzed hospital-acquired adverse events in patients undergoing lung cancer resection using the National Hospital Discharge Survey (NHDS) database from 2001–2010. Demographics, diagnoses, and procedures data were abstracted using ICD-9 codes. We used the Agency for Healthcare Research and Quality (AHRQ) Patient Safety Indicators (PSI) to identify hospital-acquired adverse events. Weighted analyses were performed using t-tests and chi-square.

**Results:**

A total of 302,444 hospitalizations for lung cancer resection and were included in the analysis. Incidence of PSI increased over time (28% in 2001–2002 vs 34% in 2009–2010; *P*<0.001). Those with one or more PSI had increased in-hospital mortality (aOR = 11.1; 95% CI, 4.7–26.1; *P*<0.001) and prolonged hospitalization (12.5 vs 7.8 days; *P*<0.001). However, among those with PSI, in-hospital mortality decreased over time, from 17% in 2001–2002 to 2% in 2009–2010.

**Conclusions:**

In a recent ten-year period, documented rates of adverse events associated with lung cancer resection increased. Despite this increase in safety events, we observed that mortality decreased. Because such metrics may be incorporated into hospital rankings and reimbursement considerations, adverse event coding consistency and content merit further evaluation.

## Introduction

Recent decades have seen considerable advances in the surgical management of cancer, including minimally invasive approaches, improved imaging and staging, and increased coordination of peri-operative care. For lung cancer, specific developments include growing use of positron emission tomography-computed tomography (PET-CT) for pre-operative staging, video-assisted thoracic surgery (VATS), and robotic approaches. Several studies have demonstrated favorable effects on outcomes, including fewer “futile” thoracotomies, better post-operative pain control and function, and reduced length of hospitalization [1–3]. Focusing on specific surgical complications [4, 5], surgical database studies have demonstrated that mortality, morbidity and duration of hospital stay have improved with contemporary surgical practices [6, 7].

In the contemporary era of heightened attention to safety and quality, we have also witnessed an increase in recognition and reporting of adverse events [8–10]. Indeed, tracking complications of medical treatment is no longer relegated to the domain of research studies, but is incorporated into real-time evaluation of care [11, 12]. Accompanying this trend has been progress towards standardization of event tracking, with financial and clinical measures broadly applied across hospitalizations and institutions, regardless of admitting diagnosis or inpatient procedures [13–15].

In turn, medical systems and payors track these metrics, with particular focus on preventable events [16–18]. Because these data may be incorporated into facility ratings and reimbursement, they have real-time and practical implications for the treatment of lung cancer and other malignancies. We therefore analyzed a widely used and validated measure of care quality and safety, Agency for Healthcare Research and Quality (AHRQ) Patient Safety Indicators (PSI)[19], in a representative national dataset, the National Hospital Discharge Survey (NHDS).

## Methods

### Data source

The NHDS is a national survey conducted annually from 1965–2010 that reports discharge data from non-federal short-stay hospitals. Abstracted data includes diagnosis codes, demographics, procedure codes, in-hospital mortality, and length of stay. The NHDS has been used in multiple previous studies to report trends and outcomes representative of the U.S. population [20–23].

### Data collection

This study was exempt from IRB review. As has been previously described [23], we analysed hospital-acquired adverse events in hospitalizations for lung cancer resection using the National Hospital Discharge Survey (NHDS) database from 2001–2010. We selected this time period because it is the most recent available in the NHDS and therefore the most representative of contemporary clinical practice. We identified lung cancer cases using the International Classification of Diseases, Ninth Revision (ICD-9) diagnosis code 162.X (malignant neoplasm of trachea, bronchus, lung). We identified lung resections using procedure codes 32.3X (segmental resection of the lung), 32.4X (lobectomy resection of the lung), 32.5X (complete pneumonectomy), and 32.9 (other excision lung). Wedge resections were not individually coded in the ICD-9. We collected demographic information including age, sex, race, and admission type. We analyzed comorbidities using individual disease ICD codes and the Charlson comorbidity index. As described previously [23], we determined AHRQ PSI using ICD-9 codes to identify hospital-acquired adverse events (Table 1) [24]. AHRQ PSI have been previously associated with length of stay and mortality for hospital admissions [25].

**Table 1:** Agency for Healthcare Quality and Research (AHRQ) Patient Safety Indicators (PSI) and corresponding ICD-9 codes.

Category	Patient Safety Indicator	ICD-9-CM
Surgical	Anesthetic complications	E8763, E9381, E9382, E9383, E9384, E9385, E9386, E9387, E9389, 9681, 9682, 9683, 9684, 9687, E8551
	Foreign body	9984, 9987, E871x
	Iatrogenic pneumothorax	5121
	Post-operative hip fracture	820xx
	Postoperative hematoma or hemorrhage	9981x, 388x, 3941, 3998, 4995, 5793, 6094, 1809, 540, 5412, 6094, 5919, 610, 6998, 7014,7109,7591, 7592, 8604
	Postoperative wound dehiscence	5461
	Accidental puncture or laceration	E870x, 9982
Healthcare associated infection	Central venous catheter-related blood stream infection	99662, 9993, 99931, 99932
	Postoperative sepsis	038x, 038xx, 9980x, 9959x
Medicine	Postoperative physiological and metabolic derangement (secondary diabetes or acute kidney failure)	249x, 2501x, 2502x, 2503x, 584x, 586, 9975
	Postoperative physiological and metabolic derangement (dialysis)	3995, 5498
	Postoperative deep vein thrombosis or pulmonary embolism	4511x, 4512, 45181, 4519, 4534x, 4538, 4539, 4151x
	Transfusion reaction	9996x, 9997x, E8760

### Statistical analysis

We summarized demographic and clinical data using frequencies and percentages for categorical variables and means or medians for continuous variables. All analyses were performed using SAS version 9.4 (SAS Corporation, Cary, NC). Weighted analyses were used in all analyses to obtain nationwide estimates and to account for the stratified sampling process of the NHDS database.

We used t-tests and chi-square tests to compare medians and proportions in hospitalizations with or without PSI that resulted in death or discharge. Weighted logistic regression was used for analysis of in-hospital mortality. Length of stay was analyzed using weighted linear regression. Multivariable models were used to calculate *P*-values adjusted for clinical characteristics that were significantly different between those with and without a PSI. A *P* value less than 0.001 was considered statistically significant. When appropriate, continuous variables were reported as mean ± standard deviation (SD) or median ± interquartile range (IQR). Categorical variables were reported as percentages and compared using odds ratios (OR) and adjusted ORs (aOR), with 95% confidence intervals (CI).

## Results

A total of 302,444 hospitalizations were included in the analysis; 52% were female, 57% older than 65 years, and 66% were white. Additional demographic information is presented in Table 2. Age, sex, race, admission type, facility type, year of resection, Charlson index, and geographic region were not associated with risk of PSI. Specific comorbidities of pulmonary circulatory disease, renal failure, and hypertension were associated with increased PSI risk.

**Table 2:** Baseline demographics and clinical characteristics in the overall cohort and by occurrence of Patient Safety Indicators.

Characteristic	PSI N (%)	No PSI N (%)	Total N (%)	*P* value	Adjusted *P* value^¶^
**Total**	88044	214400	302444		
**Age (years)**					
18–49	4236 (5)	12100 (6)	16336 (6)	0.51^†^	0.43
50–65	32502 (37)	81436 (38)	113938 (38)		
66–79	39915 (45)	100641 (47)	140556 (49)		
≥80	11391 (13)	20223 (9)	31614 (10)		
**Year**					
2001	6019 (7)	18452 (9)	24471 (8)	0.64^†^	0.33
2002	9989 (11)	23376 (11)	33365 (11)		
2003	7585 (9)	20529 (10)	28114 (9)		
2004	6117 (7)	20522 (10)	26639 (9)		
2005	7579 (9)	20018 (9)	27597 (9)		
2006	10367 (12)	24390 (11)	34757 (12)		
2007	6657 (8)	21282 (10)	27939 (9)		
2008	10148 (12)	20442 (10)	30590 (10)		
2009	12157 (14)	26772 (13)	38929 (13)		
2010	11426 (13)	18617 (9)	30043 (10)		
**Sex**					
Male	46408 (53)	99866 (47)	146274 (48)	0.13^†^	0.18
**Race**					
White	58052 (66)	140997 (66)	199049 (66)	0.39^†^	0.22
African American	3602 (4)	14667 (7)	18269 (6)		
Asian	1498 (2)	4942 (2)	6440 (2)		
Others	1094 (1)	3790 (2)	4884 (2)		
Not Stated	23798 (27)	50004 (23)	73802 (24)		
**Geographic Region**					
Northeast	19816 (23)	54438 (25)	74254 (25)	0.83^†^	0.87
Midwest	18218 (21)	45137 (21)	63355 (21)		
South	31545 (36)	74213 (35)	105758 (35)		
West	18465 (21)	40612 (19)	59077 (20)		
**Facility characteristics**					
**Number of beds**					
6–99	3042 (4)	5871 (3)	8913 (3)	0.17^†^	0.15
100–199	12830 (15)	32507 (15)	45337 (15)		
200–299	24961 (29)	47892 (22)	72853 (24)		
300–499	31830 (36)	74935 (35)	106765 (35)		
500+	15381 (18)	53195 (25)	68576 (23)		
**Hospital Ownership**					
Proprietary	6408 (7)	13030 (6)	19438 (6)	0.11^†^	0.084
Government	6886 (8)	26930 (13)	33816 (11)		
Nonprofit, including church	74750 (85)	174440 (81)	249190 (82)		
**Type of admission**					
Elective	70711 (80)	167412 (78)	238123 (79)	0.40^†^	0.35
Emergency/Urgent	9691 (11)	21728 (10)	31419 (10)		
NA	7642 (9)	25260 (12)	32902 (11)		
**Charlson comorbidity index**					
Median (Q1, Q3)	2.7 (2.0, 5.4)	2.5 (2.0, 3.9)	2.5 (2.0, 4.0)	0.24^‡^	0.16
Range	2.0–12.0	2.0–12.0	2.0–15.0		
**Specific comorbidities**					
Acquired immune deficiency syndrome	0 (0)	221 (0.1)	221 (0.1)	0.99^§^	DNC**
Alcohol abuse	1516 (2)	6330 (3)	7846 (3)	0.37^†^	0.29
Deficiency Anemias	3920 (5)	10336 (5)	14256 (5)	0.81^†^	0.65
Rheumatoid arthritis/collagen vascular disease	1596 (2)	3271 (2)	4867 (2)	0.75^†^	0.77
Chronic blood loss anemia	19 (0)	1513 (1)	1532 (1)	< .001^†^	0.003
Congestive heart failure	6135 (7)	6841 (3)	12976 (4)	0.016^†^	0.045
Chronic pulmonary disease	39822 (45)	84635 (40)	124457 (41)	0.15^†^	0.089
Coagulopathy	933 (1)	2935 (1)	3868 (1)	0.71^†^	0.61
Depression	1703 (2)	9489 (4)	11192 (4)	0.16^†^	0.37
Diabetes w/o chronic complications	5523 (6)	22625 (11)	28148 (9)	0.032^†^	0.11
Diabetes w/ chronic complications	1461 (2)	1435 (1)	2896 (1)	0.25^†^	0.40
Drug abuse	368 (0.4)	2329 (1)	2697 (1)	0.21^†^	0.26
Hypothyroidism	2918 (3)	13798 (6)	16716 (6)	0.14^†^	0.051
Liver disease	1258 (1)	1978 (1)	3236 (1)	0.63^†^	0.82
Lymphoma	20 (0)	1407 (1)	1427 (1)	< .001^†^	<0.001
Fluid and electrolyte disorders	11044 (13)	18301 (9)	29345 (10)	0.077^†^	0.074
Metastatic cancer	21112 (24)	47478 (22)	68590 (23)	0.59^†^	0.30
Other neurological disorders	888 (1)	1522 (1)	2410 (1)	0.71^†^	0.93
Obesity	1129 (1)	7746 (4)	8875 (3)	0.067^†^	0.26
Paralysis	301 (0.3)	1597 (1)	1898 (1)	0.44^†^	0.50
Peripheral vascular disease	2862 (3)	11519 (5)	14381 (5)	0.18^†^	0.15
Psychoses	413 (1)	1709 (1)	2112 (1)	0.38^†^	0.48
Pulmonary circulation disease	843 (1)	574 (0.3)	1417 (1)	0.020^†^	0.033
Renal failure	3334 (4)	1689 (1)	5023 (2)	<0.001^†^	0.004
Solid tumor w/out metastasis	7157 (8)	7770 (4)	14927 (5)	0.019^†^	0.008
Peptic ulcer disease bleeding	0 (0)	0 (0)	0 (0)	1.00^§^	DNC**
Valvular disease	1168 (1)	8159 (4)	9327 (3)	0.002^†^	0.008
Weight loss	3780 (4)	3483 (2)	7263 (2)	0.081^†^	0.25
Hypertension	3452 (4)	5507 (3)	6353 (2)	0.006^†^	0.002

Numbers indicate N (%) unless otherwise noted

^†^Chi-square

^‡^Wilcoxon

^§^Fisher exact

^¶^Logistic regression, adjusted for age, year, type of admission, acquired immune deficiency syndrome, anemias, diabetes without complications, drug abuse, pulmonary circulation disease, renal failure, solid tumor w/out metastasis, and hypertension

**Model did not converge; p-value cannot be calculated.

DNC, did not calculate

Overall, PSI occurred in 29% of cases. The rate increased significantly over time, from 28% in 2001–2012 to 34% in 2009–2010 (*P*<0.001). Rates of several individual PSI increased during this period: postoperative respiratory failure (11.6% to 14.5%), secondary diabetes or acute kidney failure (1% to 6%), postoperative sepsis (0.8% to 2.2%). Incidence of iatrogenic pneumothorax remained relatively constant at approximately 15% over the study period. Incidence of specific PSI differed between resection type (Table 3). Pneumonectomy was associated with higher rates of sepsis and respiratory failure compared to segmentectomy and lobectomy. In contrast, pneumonectomy had a lower incidence of pneumothorax, compared to segmentectomy and lobectomy, which likely reflects the removal of all ipsilateral pulmonary tissue. Incidence of overall PSI did not vary according to type of resection (Table 4).

**Table 3:** Incidence of specific Patient Safety Indicators occurring in ≥1% of 302,444 hospitalizations for lung cancer surgery.*.

Patient safety indicator	Overall N (%)	Lobectomy N (%)	Segmentectomy N (%)	Pneumonectomy N (%)
Any	88044 (29)	72273 (30)	8478 (24)	7221 (30)
Iatrogenic pneumothorax	44516 (15)	38879 (16)	4937 (14)	656 (3)
Postoperative respiratory failure	30390 (10)	23681 (10)	2649 (8)	4016 (17)
Secondary diabetes or acute kidney failure	10988 (4)	8714 (4)	776 (2)	1454 (6)
Postoperative hemorrhage or hematoma	6437 (2)	3769 (2)	1462 (4)	1206 (5)
Postoperative sepsis	3612 (1)	2742 (1)	144 (0.4)	726 (3)

*The following AHRQ PSI each occurred in less than 1% of hospitalizations: accidental puncture or laceration, anesthetic complication, central venous catheter-related blood stream infection, dialysis, foreign body, hip fracture, postoperative deep vein thrombosis or pulmonary embolus, postoperative wound dehiscence, pressure ulcers, and transfusion reaction.

**Table 4:** Comparison of overall Patient Safety Indictors between resection types.

	PSI N (%)	No PSI N (%)	Odds Ratio (95% CI)	*P* value	Adjusted* Odds Ratio (95% CI)	Adjusted* *P* value
Lobectomy	72,273 (29.8)	170,268 (70.2)	1.34 (0.85, 2.13)	0.89	1.27 (0.80, 2.03)	0.85
Pneumonectomy	7,221 (29.7)	17,132 (70.3)	1.34 (0.70, 2.56)	0.89	1.31 (0.66, 2.61)	0.95
Segmentectomy	8,478 (24.0)	26,892 (76.0)	Reference		Reference	

* Adjusted for age, year, gender, blood loss, CHF, diabetes without complications, lymphoma, pulmonary circulation disease, renal failure, valvular disease, solid tumor w/out metastasis, and hypertension.

**Fig 1:**
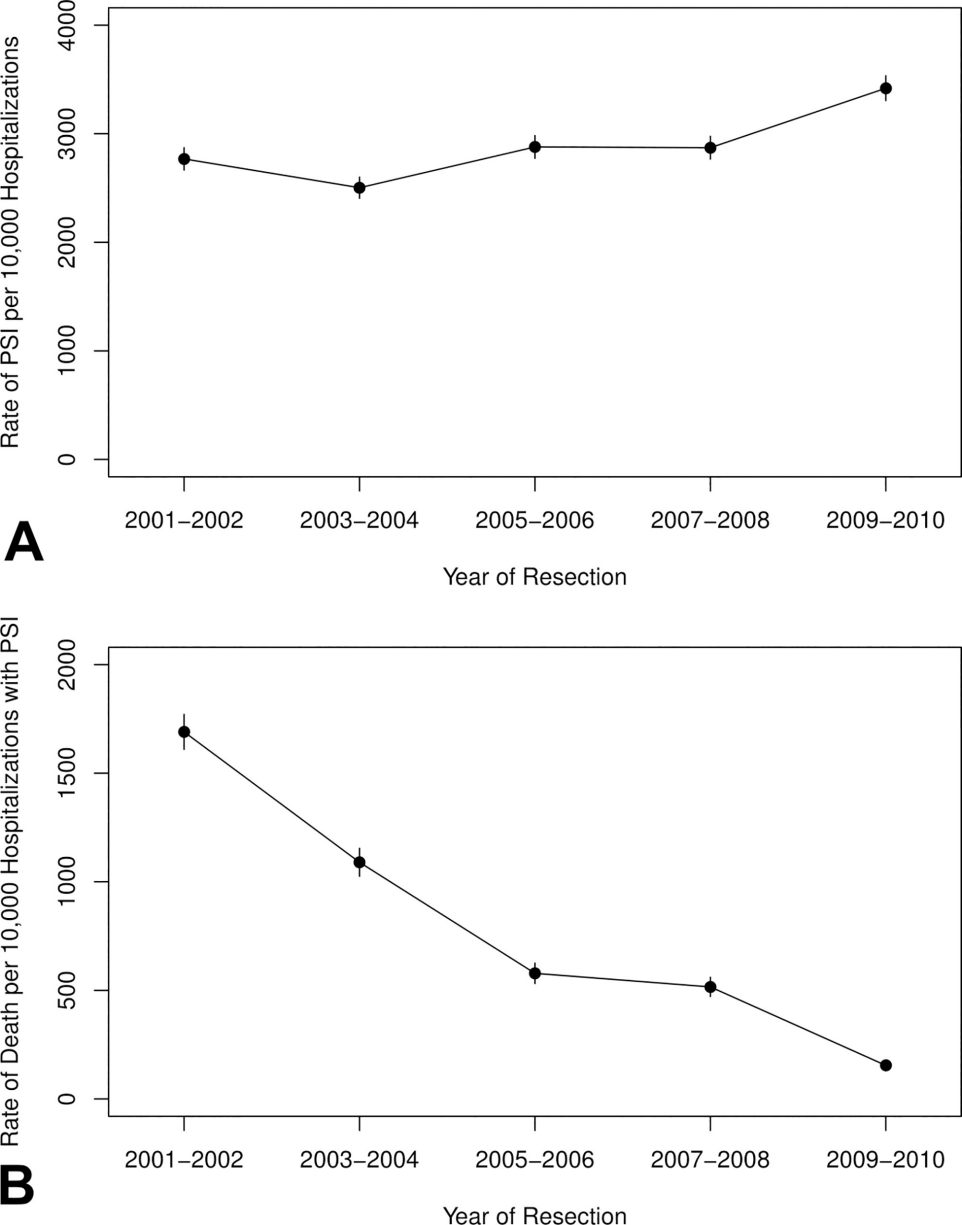
Rates of PSI and mortality with PSI over time.

PSI occurrence was significantly associated with inferior clinical outcomes. Hospitalizations with one or more PSI had a mean length of hospital stay of 12.5 days, compared to 7.8 days for hospitalizations without PSI (adjusted *P*<0.001). In-hospital mortality was 7.3% for hospitalizations with one or more PSI, versus 1% for those without PSI (adjusted OR 11.07; 95% CI, 4.69–26.12; *P*<0.001). However, among those with PSI, in-hospital mortality decreased over time, from 17% in 2001–2002 to 2% in 2009–2010 (Fig 1).

## Discussion

In this analysis of more than 300,000 U.S. hospitalizations for surgical resection of lung cancer over a recent ten-year period, we found that reported hospital-acquired adverse events—defined according to AHRQ PSI—increased by more than 20%. Although rates of events most likely directly linked to surgery, such as post-operative respiratory failure and sepsis, saw a modest increase, the greatest rise was seen in the composite event of secondary diabetes or acute kidney failure, which may have resulted from surgical or peri-operative interventions, or may have occurred secondary to other in-hospital causes. Notably, advanced age, overall comorbidity burden, chronic pulmonary disease, diabetes, and peripheral vascular disease were not associated with increased PSI. Also of note, the effect of PSI on mortality decreased over time, from 17% in 2001–2002, to 2% in 2009–2010.

There are a number of potential explanations for these findings. First, improvements in surgical techniques and peri-operative care may be leading to expanded candidacy for lung cancer surgery to include older and frailer patients, resulting in increased PSI risk. Second, earlier recognition and improved management of PSI lessen detrimental clinical effects. Finally, there could be increased recognition, documentation and reporting of PSI over time, with no associated increased rates in actual clinical adverse events, leading to an apparent reduction in associated mortality.

Elderly patients are increasingly being considered for NSCLC surgical intervention, and some studies suggest increased rates of complications and mortality after surgical intervention [26, 27]. However, as noted in other analyses [28, 29], we did not notice a significant increase in PSIs in the oldest patients, suggesting that age alone should not determine treatment exclusions, which should be based on other factors such as physiological status. Additionally, patients with increasing comorbidity burden have increased risk of postoperative complications after lung cancer surgery [30]. In one study conducted during the era of the present analysis, up to 50% of patients undergoing lung cancer resection had at least three comorbidities [31]. However, in the current study, age and most comorbidities were not associated with increased PSI risk, rendering this explanation less plausible.

Over time, improvements in peri-operative care have led to enhanced prevention, recognition, and management of surgical complications. For lung cancer resection, these measures include, among others, chest physiotherapy, airway suctioning, mucolytics, and high-flow nasal canula [32]. Additionally, during the period of this study, improvements in recognition and treatment of sepsis, acute kidney injury and venous thromboembolism emerged [33–40]. To what extent these developments underlie the increased incidence but decreased significance of PSI in the present study is not clear.

Alternatively—in the contemporary era of heightened vigilance, attention to patient safety, and documentation—it seems plausible that clinical events, including PSI, are being recorded more frequently, even if the actual rate has not risen. The temporal increase in composite rate of adverse events in our study directly contrasts findings from the thoracic surgery literature, which report improvements in near- and long-term clinical outcomes [41–43]. A previous analysis of all cancer surgeries in the Nationwide Inpatient Sample (NIS) database also found that reported hospital-acquired adverse events are increasing over time [44]. Despite substantial differences between the databases [45], our findings are consistent with the findings of this previous analysis [44]. Supporting this hypothesis is the marked decrease in hospital mortality we observed in cases experiencing PSI, from 17% in 2001–2002 to 2% in 2009–2010. This behavioral trend could particularly impact complications defined somewhat subjectively, such as “respiratory failure,” a term that encompasses aspiration, pneumonia, sepsis, acute respiratory distress syndrome, chronic heart failure, and venous thromboembolic events, among others [46]. Similarly, the term “sepsis” has been subject to changes in diagnostic threshold over the years, which may influence coding patterns and have implications for epidemiological and clinical research [47]. By contrast, rates of pneumothorax and venous thromboembolic events, tend to be more objectively defined based on radiographic findings, remained relatively stable over time.

What factors would result in a possible behavioral shift in event coding practice? The “culture of quality” in healthcare, designed to improve team function, patient centeredness, transparency, and outcomes, stresses the importance of detailed and complete documentation [14, 15, 48, 49]. Alternatively, widespread implementation of the electronic health record may have simplified the process of selecting and recording diagnostic codes [50]. Whatever the dominant cause, this trend has led to concerns about the interpretability and generalizability of these data [51, 52]. For much of modern-day clinical practice, reimbursement is tightly linked to documentation. Demonstration of medical complexity may allow billing at a higher level. For instance, admitting diagnoses of “Sepsis” and “Urinary tract infection, site not specified from urinary source” (ICD-9 995.92, 599.0) results in greater compensation than “Urinary tract infection, site not specified” (ICD-9 599.0) alone. While capturing comorbidities and severity at the time of clinical intake or admission may favorably impact a facility’s rating or reimbursement, if this practice is applied to events occurring during or after hospitalization, it could have the opposite effect. Indeed, others have raised concerns that coding and documentation inconsistencies may limit the validity of employing PSI to evaluate hospital performance [19, 53, 54].

Even if rising PSI rates are due to changes in documentation practice rather than an actual increase in clinical complications, efforts to limit these events remain central to optimizing patient care. Among all major malignancies, the development of post-operative sepsis confers the greatest mortality to patients with lung cancer [55]. In a large prospective cohort study, post-operative sepsis added $26,972 in hospital costs [56]. Yet a single intervention—procedural checklists—may reduce bloodstream infections by up to 57 percent [57]. In the present study, even though the magnitude of effect decreased over time, PSI remained associated with longer hospitalization and increased mortality, suggesting that these cases continue to merit attention and intervention.

Limitations of this study include lack of data on surgical approach (eg, open, VATS, robotic-assisted), type of surgeon, type of hospital, cancer stage, or long-term outcomes. Some specific complications associated with thoracic surgery, for example, atrial dysrhythmias, are not included in AHCQ PSI evaluations. Aside from events for which the timing is inherently defined (eg, postoperative respiratory failure), PSI may have temporally preceded surgical resection and therefore not technically resulted from the procedure. In addition, the definition pneumothorax is based on ICD coding, and as such this study is unable to determine if pneumothorax is being coded due to a pneumonectomy space or if the pneumothorax coding represents true pathology in the remaining lung. Importantly, our unit of measurement is hospitalization rather than patient, as the NHDS does not have a mechanism to differentiate individual patients. Although it would be possible for a single patient to undergo multiple hospitalizations for lung cancer surgery, sequential lung cancer surgeries in separate hospitalizations are rarely performed, even in patients with multiple synchronous primary tumors [58]. We also recognize that almost a decade has passed since the end date of our cohort, during which time further advances in surgical technique and supportive care have occurred. Unfortunately, discontinuation of NHDS after 2010 precludes the analysis of more recent data. Nevertheless, we believe that the study’s large sample size, geographically and demographically diverse and representative cohort, and detailed diagnostic coding data render our findings relevant to contemporary considerations for the care of patients undergoing lung cancer resection.

## Conclusion

To our knowledge, this study reports the largest cohort of hospitalizations for lung cancer resection described to date. In contrast to a consistent thoracic surgery literature reporting improved outcomes over time, we found increasing rates of documented hospital-acquired events defined according to widely used AHRQ PSI. However, the increase in these events over time did not correlate with worse clinical outcomes. It is unclear if these findings represent changes in coding practices, patient selection, or improved recognition and treatment of complications. Nevertheless, because these metrics are considered in hospital ratings and reimbursement, further study of adverse event reporting and behavior in thoracic surgery and other populations is warranted.

## Supporting information

S1 Data (XLSX)
